# Rapid and complete paraffin removal from human tissue sections delivers enhanced Raman spectroscopic and histopathological analysis†
†Electronic supplementary information (ESI) available. See DOI: 10.1039/c9an01030k


**DOI:** 10.1039/c9an01030k

**Published:** 2019-12-19

**Authors:** Riana Gaifulina, Daren J. Caruana, Dahmane Oukrif, Naomi J. Guppy, Siân Culley, Robert Brown, Ian Bell, Manuel Rodriguez-Justo, Katherine Lau, Geraint M. H. Thomas

**Affiliations:** a Department of Cell and Developmental Biology , University College London , UK . Email: g.thomas@ucl.ac.uk ; Tel: +44 (0)20 7679 6098; b Department of Chemistry , University College London , UK; c Research Department of Pathology , University College London , UK; d UCL Advanced Diagnostics , University College Hospital , UK; e MRC Laboratory for Molecular Cell Biology , University College London , UK; f Spectroscopy Products Division , Renishaw plc , UK . Email: katherine.lau@nikon.com; g Department of Gastrointestinal Pathology , University College Hospital and Department of Research Pathology/Cancer Institute , UCL , UK

## Abstract

Incomplete removal of paraffin and organic contaminants from tissues processed for diagnostic histology has been a profound barrier to the introduction of Raman spectroscopic techniques into clinical practice. We report a route to rapid and complete paraffin removal from a range of formalin-fixed paraffin embedded tissues using super mirror stainless steel slides. The method is equally effective on a range of human and animal tissues, performs equally well with archived and new samples and is compatible with standard pathology lab procedures. We describe a general enhancement of the Raman scatter and enhanced staining with antibodies used in immunohistochemistry for clinical diagnosis. We conclude that these novel slide substrates have the power to improve diagnosis through anatomical pathology by facilitating the simultaneous combination of improved, more sensitive immunohistochemical staining and simplified, more reliable Raman spectroscopic imaging, analysis and signal processing.

## Abstract 2

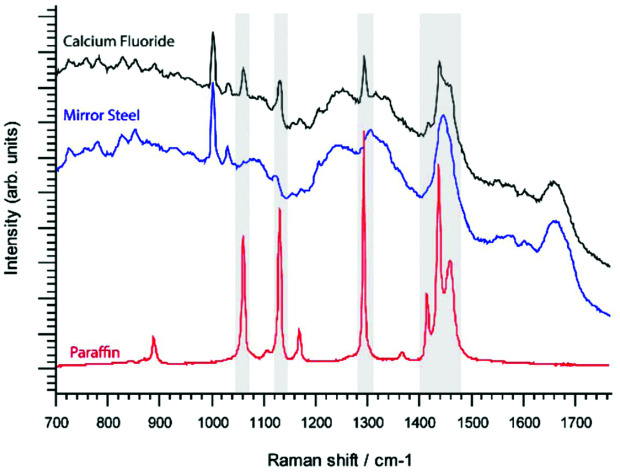
A simple method completely removes contaminating paraffin from samples for clinical Raman and enhanced immunohistological analysis.

## Introduction

The application of Raman microspectroscopy (RS) as a form of spectral histopathology has gained a great deal of popularity over the last few years.^1^,^2^ However, application of the technique for diagnostics is challenging when using the conventional formalin-fixed paraffin-embedded (FFPE) tissues employed almost universally in anatomical pathology and is widely available in tissue sample archives. This has restrained the widespread adoption of RS in pathology.^3^–^7^ As a vibrational spectroscopic technique RS provides sample-specific chemical information with few sample preparation requirements, which means that in principle RS could be easily integrated into the established pathology workflows. However, where RS and its associated technique – Infrared Spectroscopy (IR) – have been applied in pathology^8^–^10^ both techniques confirm the failure of conventional tissue processing protocols or aggressive solvent extraction regimes to completely remove paraffin contamination from FFPE sections. The remaining paraffin signals confound reliable multivariate statistical classification of disease status from the RS signals alone and, in addition, its presence has also been revealed as the cause of a substantial loss of Immunohistochemical (IHC) staining intensity in samples destined for conventional pathology analysis.^8^

Following the discovery using RS that FFPE histology sections retain paraffin contamination,^11^ investigators have attempted to achieve complete deparaffinization using a variety of known solvents. Faoláin *et al.* were first to use RS to detect paraffin in FFPE tonsil and cervical tissues and they later went on to test whether paraffin can be completely removed using an array of well-known deparaffinizing agents: xylene, Histo-Clear, heat-mediated antigen retrieval (HMAR) processes using xylene and citrate buffer, and Trilogy (combined deparaffinization and unmasking of antigens). The potential of hexane as a dewaxing agent was also investigated. They found that none of the solvents completely removed paraffin and only after 18 hours of immersion in hexane, an industrial degreasing agent, that paraffin although much reduced was still retained. However, prolonged treatment with hexane gave a 28% increase in IHC staining intensity when compared to the conventional brief xylene treatment.^8^ Clearly there is potential to improve antigen detection if paraffin can be reliably removed, however it is not known if hexane degrades other signals in tissue that might be diagnostically useful like nucleic acids or lipids, or if protracted periods of solvent extraction provide sufficient benefit for cost in pathology lab workflows.

This opportunity to greatly improve diagnostic sensitivity is not well-known in the histopathology community perhaps due to a lack of easily available and reliable methods to detect the degree of paraffin contamination. A later study by Nallala *et al.* compared the deparaffinization efficacy of xylene, hexane and paraffin oil with an additional hexane wash using IR spectroscopy.^9^ They estimated the amount of paraffin remaining after each solvent immersion by using coefficients based on the fit of a pure paraffin model. They found paraffin oil followed by a hexane wash to be superior to using xylene and hexane alone, but confirmed that neither completely removed paraffin. A variable degree of paraffin retention was also found to be dependent upon tissue architecture with connective tissue regions retaining more wax than glandular tissue. Other groups have attempted to minimise the contamination of Raman spectra with paraffin peaks using a number of computational processing approaches. This includes digital dewaxing^12^ and paraffin component isolation and subsequent subtraction, however these approaches necessitate transformation of the raw dataset which may impact on diagnostic power or reliability of disease classification downstream.^13^ As a result, the current situation is that complete removal of paraffin wax from FFPE histological sections and the Raman signals derived from them has not yet been achieved.

Here, we report experimental results revealing complete paraffin removal from tissue sections mounted onto a novel backing substrate – super mirror finish stainless steel (SS) slides. We demonstrate that enhanced IHC staining can be achieved using this substrate and propose a plausible mechanism for the paraffin removal phenomenon. In addition to IHC enhancement we also report an enhancement in the overall Raman signal compared to that achieved on the conventional CaF_2_ slides. The SS slide have other significant advantages in that they are mechanically robust and inexpensive in comparison to the standard RS CaF_2_ slides which are both very costly and brittle. The SS slides will overcome these barriers that have so far prevented the routine adoption of RS into diagnostic pathology and medicine.

## Results

### Complete paraffin removal

**Fig. 1:**
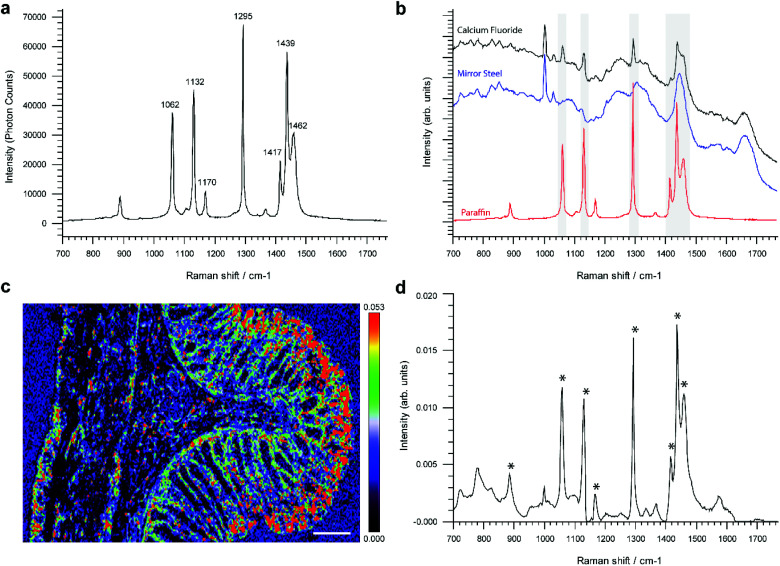
Raman analysis of paraffin contamination in FFPE tissues. a, Raman reference spectrum of histology grade paraffin wax. b, Comparison of paraffin spectrum with single point Raman spectra of 8 μm colonic tissue sections mounted on calcium fluoride (black spectrum) and super mirror steel (blue spectrum). c, Inhomogeneous distribution of typical contaminating paraffin in rat colon tissue mounted on CaF_2_. Tissue map of a paraffin derived pure MCR-ALS derived component (shown in panel d) presented in rainbow colour scale (scale bar: 200 μm). Regions with dense paraffin contamination are shown in red. d, Pure component curve extracted by MCR-ALS analysis reveals an almost identical spectral profile to a paraffin reference spectrum shown in panel A, identical paraffin peaks are marked by asterisks.

Reference single point spectra obtained from a pure paraffin sample, as well as tissues mounted onto the conventional RS imaging substrate CaF_2_ or SS slides, are shown in Fig. 1a and b. Spectra were collected from identical anatomical regions in the colon; in this case they were collected from the mucosa as this region is more structurally complex in the colon. The prominent spectral features in the sample on CaF_2_ derived from retained paraffin are due to C–C stretches and CH_2_ deformations, the latter causing the most intense paraffin peak at 1295 cm^–1^ (Fig. 1a). As shown, six out of the eight paraffin peaks clearly contaminate an averaged point spectrum acquired following xylene deparaffinized tissue mounted onto CaF_2_ (black spectrum). However, these peaks are absent from the same tissue mounted onto mirror SS (blue spectrum) (Fig. 1b).

Performing unsupervised MCR-ALS analysis on a high resolution Raman map taken across the full thickness of rat colon on CaF_2_ enabled us to reconstruct a pure component spectral profile of paraffin and identify the spatial distribution of paraffin contamination (Fig. 1c and d). We chose to use rat colon for this study as we wanted to capture a high-resolution Raman map of the full thickness of the colon wall and hence attempt to identify if paraffin adheres selectively to specific tissue types and architecture. It would have been more challenging to deliver a Raman image of the same resolution in human colon due to its significantly larger size. Results show that paraffin is predominantly localised at the luminal edge of the mucosa (Fig. 1c).

Given that paraffin appeared to selectively adhere to the mucosa in rat tissue, Raman maps were taken specifically from the mucosa and muscularis propria from human colonic tissue mounted on SS slides and CaF_2_ (ESI Fig. 1†). Using unsupervised principal component analysis we were able to demonstrate the presence of paraffin contamination only in tissue mounted on CaF_2_, there were no peaks associated with paraffin in either the mucosa or muscle regions of the colon mounted on SS (ESI Fig. 2 and 3†). Contamination however was found in both the mucosa and muscle regions of the colonic tissues mounted on CaF_2_.

Similar maps, albeit of lower spatial resolution, were taken across deparaffinized FFPE human ovaries, brain, tonsil, colon and oesophageal tissue sections mounted on mirror SS. In total, we have analysed 18 ovarian cancer tissues, 50 samples encompassing four different pathologies of paediatric brain tumours, 36 healthy human tonsils, 65 colon tissues encompassing normal and cancer and 154 oesophageal samples across normal tissue and multiple different cancer pathologies. Of all the 323 Raman maps analysed, none showed any sign of paraffin contamination following xylene washes, the results of these independent studies are yet to be published. This suggests that the properties of complete paraffin removal on mirrored SS slides are independent of the tissue type and its anatomical complexity, morphology and biochemistry. Importantly, complete deparaffinization is achieved with xylene within minutes on SS slides. We have tested a wide range of xylene incubation protocols, varying the number of washes in fresh xylene, the incubation time as well as the temperature of xylene. All combinations proved to be ineffective in completely removing paraffin contamination from tissues mounted on CaF_2_, synthetic fused silica (quartz) or standard glass, however all combinations were successful in achieving complete paraffin removal from tissues mounted on super mirror steel. As a result, we opted for a 10-minute xylene step (fresh xylene) which is comparable to standard pathology lab protocols in terms of exposure time although in our hands complete deparaffinization can be completed in less than 1 minute with a single wash if required. We also note the potential benefit of minimising the washing step in reducing extraction of non-polar biochemical components, for example lipids, which could be retained for future analysis and use as biomarkers.

### Immunohistochemical enhancement

**Fig. 2:**
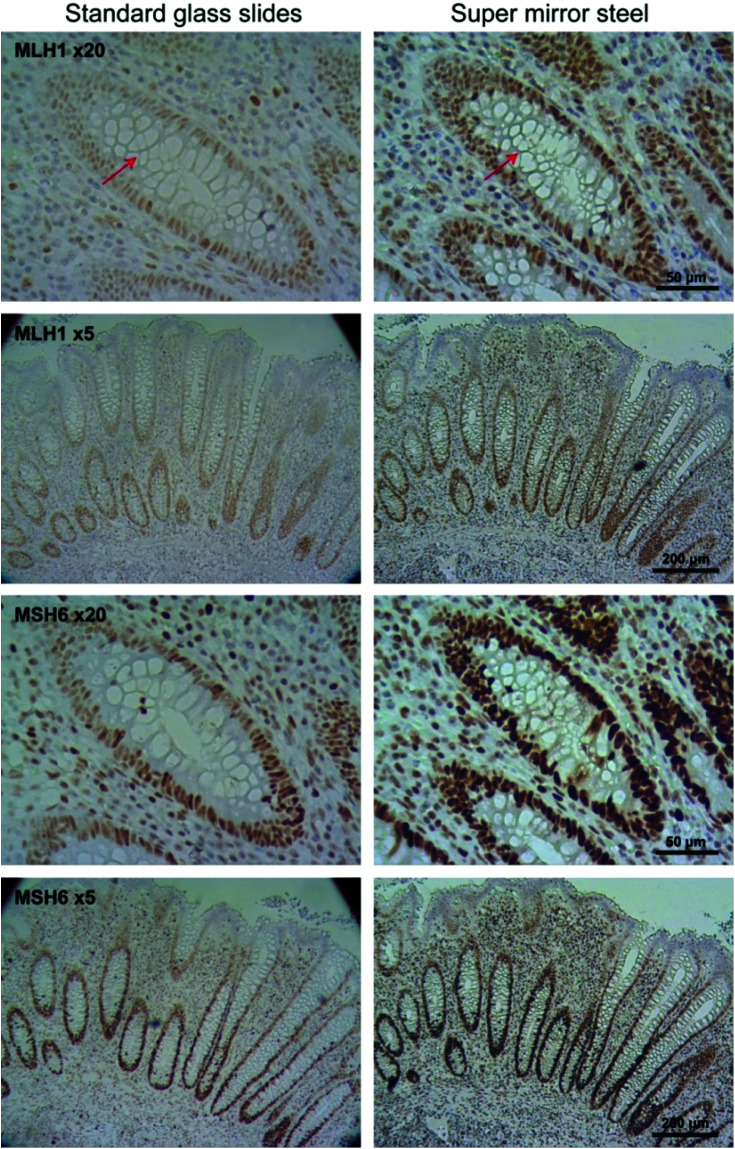
Comparison of immunohistochemical staining using MLH1 and MSH6 on conventional histology glass slides (left panels) taken using transmission microscopy and mirrored SS slides (right panels) visualised using reflectance microscopy at different magnifications (5× and 20×). MLH1 and MSH6 positive cells are stained brown with the DAB reaction.

**Fig. 3:**
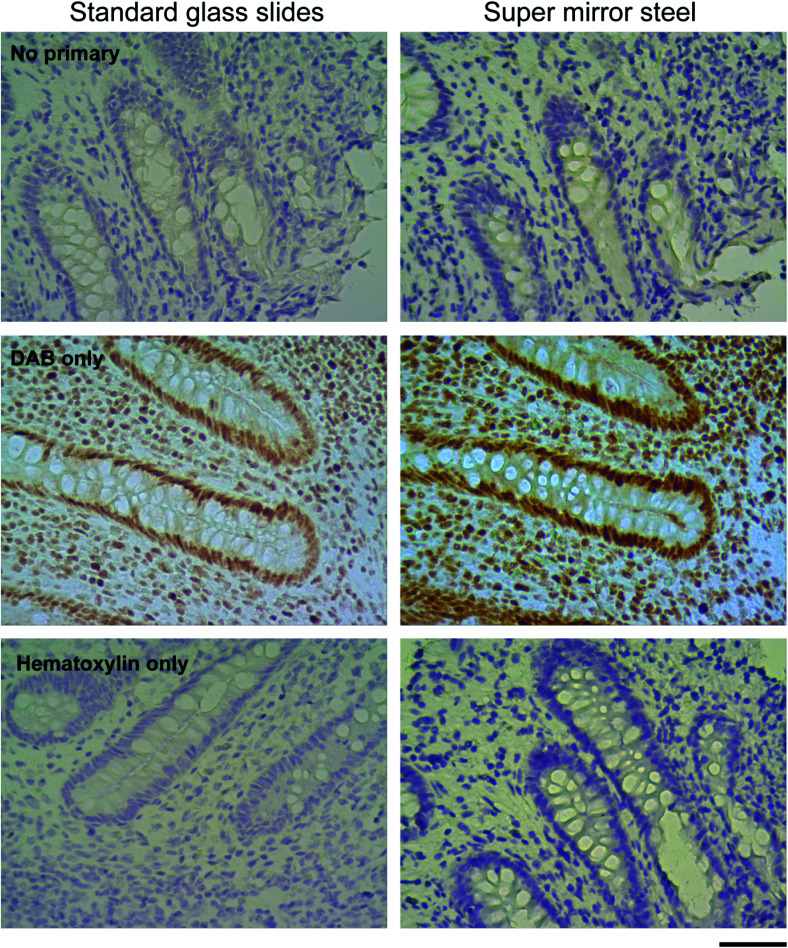
Negative control staining omitting the primary antibodies (top panels), using DAB only (middle panels) and hematoxylin only (bottom panels) to confirm the lack of any unusual interactions between the SS surface and the different steps utilised within the IHC workflow. Scale bar: 50 μm.

Immunohistochemical staining was used to determine the variability in staining intensity between tissues mounted on glass and on mirror SS in relation to paraffin removal. Antibodies against MLH1 and MSH6 DNA mismatch repair proteins were used to stain FFPE appendix tissue (Fig. 2 and 3). MLH1 and MSH6 antibodies were specifically selected for this study due to their known weak and patchy staining patterns making this a sensitive and rigorous test of the system with probes of clinical relevance.^14^ In routine clinical practice the slides are counter stained with hematoxylin (blue) to reveal cell nuclei.

Unlike conventional glass microscopy slides where sections can be easily viewed using transmission white light illumination, opaque substrates such as steel can only be viewed under *epi*-illumination (reflectance optics). Consequently, one of the main difficulties in obtaining a fair comparison between the two backing substrates is the different optical pathways used in transmitted and reflectance microscopy, as well as the use of different image acquisition settings. To ensure optimal visualisation of the tissue sections mounted on each of the substrate types, sections mounted on glass were visualised using transmission microscopy, whilst sections on SS were visualised using reflectance microscopy. In addition, IHC staining is not stoichiometric and hence it is difficult to obtain a reliable quantifiable measure of staining intensity.^15^,^16^ As a result, we did not attempt to quantify the staining intensity but instead estimated the total number of stained cells using machine learning-based image segmentation.^17^ This approach provided an automated method for investigating the potential impact of paraffin wax as a barrier between the tissue and applied histological stains and has also provided a quantitative output for direct comparison. Hence, both the 3,3′-diaminobenzidine (DAB) and hematoxylin stained cells were considered together.

Visual inspection of the IHC stains revealed that the MLH1 staining was greater in tissues mounted on mirror SS compared to glass (Fig. 2, top two panels). To confirm that the perceived staining enhancement was not caused by the nature of the reflective surface alone, visualisation of tissues mounted on glass and viewed with a super mirror steel slide placed behind the slide and visualised using *epi*-illumination was performed (ESI Fig. 4†). This arrangement did not show an increase in intensity, suggesting that the observed increase is not caused by the reflective surface. The same trend was observed for the MHS6 immunostain whereby the staining was much stronger on steel than the glass counterpart (Fig. 2, bottom two panels). There was an obvious enhancement in the image contrast, which is particularly evident around the goblet cells within the crypts (see arrows). This was confirmed visually by independent “blinded” inspection by our consultant pathologist and senior biomedical scientist – specialists trained in the visual inspection of IHC samples for diagnostic and pathology sample quality control.

To determine if the enhanced staining derives from any interaction between reagents used in the standard IHC protocols and the surfaces of the SS slides a series of control stains were created by omitting some components *e.g.* the primary antibody, the DAB reagent or hematoxylin. These protocols did not reveal any unusual interaction between the steel surface and antibodies or stains used for visualisation (Fig. 3). Control staining with DAB and hematoxylin alone but without primary antibody (No primary, Fig. 3) showed that the blue hematoxylin staining was much darker on SS slides. The absence of any brown staining provided reassurance that no false positive antigen detection arises from unexpected interactions between the SS slide surface, the DAB reagent and hematoxylin. Using the antibody and DAB alone, without hematoxylin, (DAB only, Fig. 3) revealed that the enhanced nuclear staining is not due to the counter staining (both MLH1 and MSH6 antigens are localised in the nucleus). Lastly, use of the counter stain alone without primary antibody or DAB (Hematoxylin only, Fig. 3) again confirmed the much denser colouration of the tissue on SS slides indicating that simple chemical staining is improved as well as IHC staining.

**Fig. 4:**
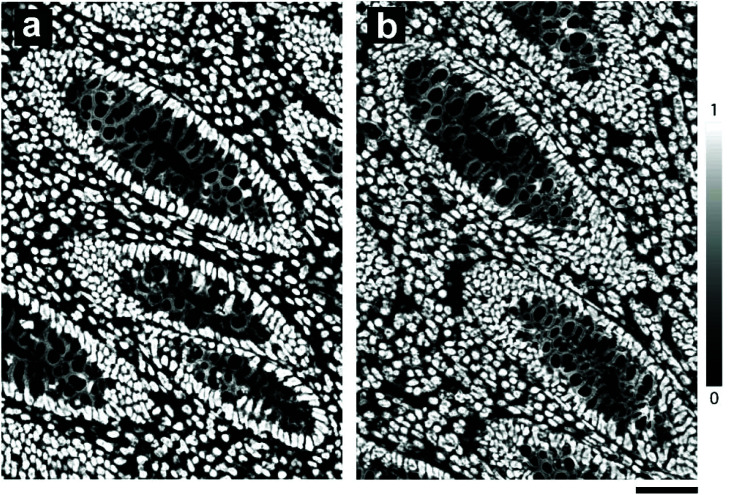
Image segmentation performed using Trainable Weka Segmentation on a small magnified region of the mucosa. a, Probability maps generated for nuclei from tissues mounted on glass, b, probability maps generated for nuclei from tissues mounted on super mirror SS. Scale bar: 50 μm.

Segmentation of nuclei from the magnified images shown in Fig. 2 indicated a total cell count of 978 on glass and 1169 on mirror SS; an estimated 20% increase in the total number of cells stained (Fig. 4). Furthermore, using the mirrored SS did not have any adverse effect on immunostaining: a Lynch Syndrome colorectal cancer resection with loss of MLH1 immunostain (case confirmed as MLH1 germline mutation by Sanger sequencing) was seen to have achieved comparable staining results to glass without compromising the diagnosis. In addition to superior staining performance on SS slides, we also observed that using a standard mounting agent and cover slipping facilitates prolonged storage of previously stained tissues mounted on SS slides. This in turn facilitates the archiving of slides as is normally done with standard glass slides following pathological review.

### Raman signal enhancement

**Fig. 5:**
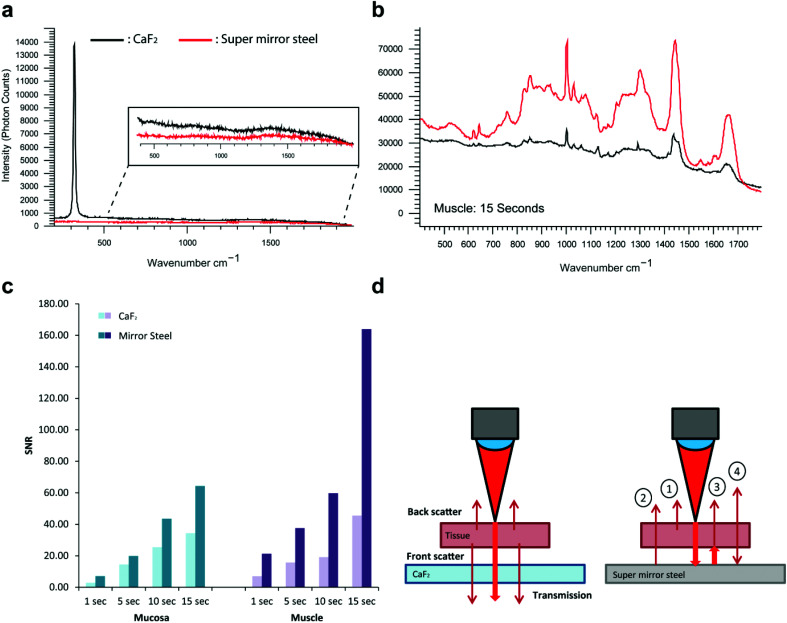
Performance comparison of conventional CaF_2_ Raman imaging slides against super mirror SS. a, Raman background spectra of CaF_2_ and super mirror SS. b, Raman tissue spectra acquired for 15 seconds from FFPE 8 μm muscle tissue section (black spectrum = CaF_2_, red spectrum = SS). c, Signal to noise ratios acquired from muscle and mucosa regions of colonic tissue sections mounted on glass and SS with varying integration times. d, Diagrammatic representation of the Raman enhancement from tissue mounted onto super mirror steel slides compared to a transparent backing substrate. Thicker red arrows depict the incident laser light and the thinner red arrows depict spontaneous Raman scatter from the tissue. (1) Backscattered Raman from incident laser excitation, (2) front scattered Raman that is normally lost is reflected back into the collected optics (3) front scattered Raman from reflected laser excitation, (4) backscattered Raman from reflected laser excitation is reflected back into the collection optics.

In addition to exhibiting superior histological performance, the mirrored SS slides are also exceptionally well suited for Raman analysis, giving a background signal that is much lower than that of the more expensive, less robust and less mechanically durable counterpart UV grade CaF_2_ used widely in the field (Fig. 5a).^18^,^19^ Reliable mounting and deparaffinization of FFPE tissue sections on the steel surface at first proved difficult, as at times the solvents detached the samples. However, this was remedied by the application of a thin 3-aminopropyltriethoxysilane (APES) coating to the steel surface in a process known as silanization.^20^ Out of all the surface treatments available, such as poly-l-lysine that facilitates tissue adhesion through a charge interaction between the tissue and slide, treatment with APES was the only surface treatment deemed to be most chemically compatible with the steel surface. This is thought to be because the metal surface hydroxyl groups react with water activated-APES to form Si–O–Cr covalent bonds on the stainless steel surface. The interaction between the tissue and slide surface is through a covalent bond and is therefore deemed to be superior to a charge-only interaction. A comparison of the steel surface was made before and after APES coating to confirm the absence of an APES Raman spectral signature.

After ensuring complete, reliable tissue adhesion, we acquired point spectra at gradually increasing signal integration times across the mucosa and muscle regions of FFPE colonic tissue. The tissue was checked post-measurement and confirmed to not have sustained any photo-thermal degradation during any of the measurements taken. A substantial signal enhancement was generally observed on mirrored SS but more so from the muscle tissue regions with the longest integration time (Fig. 5b). The magnitude of the enhancement is shown to increase with the acquisition time, with a maximum enhancement of 3.6 times and this also appears to be dependent on the composition and overall behaviour of the tissue (Fig. 5c). Unlike the mucosa which contains a heavy collagenous component, the muscle layer is predominantly composed of muscle cells, hence the muscle region will be more transparent resulting in a maximum signal enhancement that can in principle reach a theoretical upper bound of four-fold.^19^ The enhancements seen here are likely to be a result of a ‘double pass’ effect whereby the laser effectively passes through the tissue twice as it is reflected from the slide surface (Fig. 5d). This would therefore enable the collection of front scattered Raman that is often lost through transparent substrates. In principle, this should enhance the Raman signal by a factor of up to four, because front and backscattered Raman from the incident laser excitation is combined with the front and backscattered Raman originating from the reflected laser excitation. This is in accord with a previous study by Kamemoto *et al.* that used cervical tissue mounted on front coated aluminium mirrors.^19^ Considered in terms of speed, this improvement in signal acquisition (3.6 fold in these studies) will significantly reduce the time required for Raman imaging of tissue samples (here 72%) greatly enhancing the potential of RS for routine tissue analysis and diagnosis.

We also note that if limited by tissue damage we would expect to lose half of the advantage, *i.e.* be forced to reduce the laser power as the mirrored surface enhances the laser power density in the tissue by up to two-fold. On the other hand, SS might provide a better heat sink and therefore facilitate better heat transfer away from the interaction region, which is certainly a mitigating factor.

The problems of sample heating and subsequent burning caused by the double-pass of the laser could necessitate reducing the laser power during measurement. However in our hands the efficient heat sink properties of a metal slide compensate for this (with the possible exception of heavily pigmented or extensively dehydrated tissues) allowing faster, measurements.

### The mechanism for paraffin removal

We carried out a number of different experiments to elucidate the possible mechanism behind the complete paraffin removal observed from SS slides. It was initially postulated that the surface profile and overall roughness of the surface may dictate the completeness of paraffin removal. The surface profile was investigated using scanning electron microscopy (SEM) and atomic force microscopy (AFM) (ESI Fig. 5 and 6†). The roughened profile and obvious scratches seen from the high magnification of the CaF_2_ surface was a direct result of reusing and hence cleaning of the CaF_2_ slide (ESI Fig. 5d†). This practice is unavoidable in most laboratories because of the high cost of this slide substrate. The overall surface roughness of SS was very similar to that of standard microscopy slides – also known to retain paraffin wax like CaF_2_ (ESI Fig. 6†). As a result, the surface topography and roughness did not allude to a potential mechanism for complete deparaffinization. Contact angle measurements with xylene were then taken to ensure the wettability of the SS surface was similar to that of the glass, silica and CaF_2_, which was proven to be the case and therefore the rheological properties of xylene on the different substrates is unlikely to provide an explanation.

The possibility of electrochemical effects playing a role in paraffin removal arose when it was observed that the paraffin tissue section ribbon could be visibly repelled from the SS during mounting. Parreira and Schulman have previously reported that paraffin wax has a net negative charge above pH 5,^21^ subsequent pH measurements of xylene indicate a neutral environment during deparaffinization. The zeta potential of SS is reported to be negative at neutral and alkaline pH.^22^,^23^ We can therefore hypothesize that the negative charge carried by the SS surface and paraffin wax in an insulating xylene environment facilitates repulsion from the surfaces of the slide and the tissue. Previous work has shown that the work function property of metals is directly correlated to the surface charge or zero charge (pzc). A low metal work function correlates to a negative surface charge, whereas a high work function results in a positive surface charge on the dielectric after contact.^24^

To test whether metals with different work functions have different paraffin removal properties, a range of metals were mounted with 8 μm tissue sections and deparaffinized as before. Table 1 illustrates the paraffin retention properties from metal surfaces with variable work functions. There appears to be a threshold work function below which complete paraffin removal is possible, whereas a work function above this threshold value facilitates paraffin retention. The natural charge the electrode acquires which determines the surface charge, is reflected in the potential of zero charge (pzc). The pzc is a combination of the absolute electrode potential, dictated by the electrode material and the surface potential in a given electrolyte, which will determine the surface electrical charge of the metals with respect to the solution. To confirm the relation between work function and pzc in liquid, experiments outlined by Peretz *et al.* were then carried out to obtain an estimate of the pzc for each of the metals in aqueous electrolyte.^25^ These potentiostatic experiments allowed us to gain an insight into the polarity of the surface relative to the electrolyte.

**Table 1:** Summary of paraffin retention on metal surfaces with variable work function values (eV). The potentials of the determined pzc from the electrochemical measurements shown in Fig. 6

Metal	Work function (eV)	Paraffin detected (Y/N)	pzc (V *vs.* Ag/AgCl)
Aluminium coated glass	4.06–4.26	No	—
Titanium	4.33	No	–0.145 ± 0.01
**Stainless steel (304L)**	**4.40**	**No**	**–0.196 ± 0.01**
Iron	4.67–4.81	No	–0.610 ± 0.01
Platinum	5.12–5.93	Yes	+0.336 ± 0.01
Gold	5.1–5.47	Yes	+0.027 ± 0.01

**Fig. 6:**
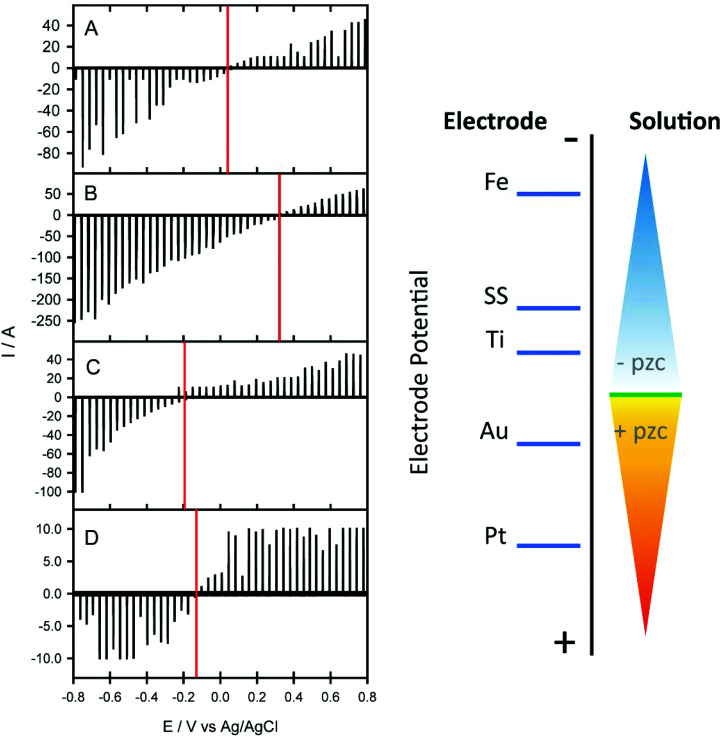
Charging current–potential traces for gold (A), platinum (B), titanium (C) and 304L stainless steel (D) obtained using KCl electrolyte solution. The measurement is based on a dropping electrolyte onto the surface of the electrode and instantaneously making contact with a counter and reference electrode to create an electrochemical cell to measure the non-faradaic charging current, the method outlined in ref. 25. The right panel depicts a diagrammatic representation of the changing surface electrode potential relative to the electrolyte solution of the different metals tested.

All of the metals shown in Table 1 that did not retain paraffin following deparaffinization (titanium, iron, stainless steel) had a negative pzc (Fig. 6) with respect to that for gold and platinum which did retain paraffin. This indicates that in a neutral environment such as that of xylene, the surface of SS will possess a net negative surface charge which can facilitate the repulsion of paraffin wax, also known to have a negative charge above pH 5. It would be desirable to make these measurements in xylene, however, due to the low solubility of electrolytes it was not possible to make any meaningful measurement. Nevertheless, from the determined relative pzc for each metal, we can extrapolate the potential where the surface charge switches from negative to positive in the xylene paraffin removal experiments. We can deduce that the xylene potential lies somewhere between the pzc of gold and titanium, to ensure that the surface of the SS is negative with respect to the xylene solution. We conclude that it is by this electrochemical effect that all paraffin is removed from the tissue sections.

Given that aluminium foil has previously been shown to be a suitable substrate for Raman imaging and has also been shown by us to undergo complete paraffin removal in xylene (Table 1), aluminium foil or coated glass slides were not deemed as suitable for Raman imaging in pathology when compared to stainless steel.^26^ Implementation of using foil alone is not feasible in routine histopathological practice as its flexibility would not be able to retain the structural integrity of the tissue under investigation, as well as generally being hard to handle. Inspection of a more structurally stable aluminium coated glass alternative not only showed an intense broad feature around 750 cm^–1^ attributed to the stretching vibration of the AlO_3_ coating, but signals from the glass under the coating became more evident when used with thinner tissue sections at 785 nm excitation (ESI Fig. 7†).

## Discussion

Using our novel APES-coated super mirror SS slides we were able to completely overcome a number of key issues encountered when using Raman spectroscopy in diagnostic pathology. One of the greatest benefits of the SS substrates reported here lies in the complete paraffin removal that was observed across a range of different tissues. Although the exact mechanism underpinning this unique deparaffinization property of SS slides is currently unknown, we hypothesize that it is caused by surface electrochemical effects as demonstrated by our experiments. Subsequently, we were able to demonstrate that complete paraffin removal leads to an increase in the global staining intensity during IHC analysis, as well as an increase in the total number of cells stained. Although this needs to be confirmed by optimisation of antibody staining on steel as well as testing a larger cohort of variable tissues, we believe that this will be diagnostically significant. The role of residual paraffin retention in reducing the global or localised efficacy of antibody staining is largely ignored in anatomical pathology because until recently it has not been possible to image easily and quantify paraffin retention *i.e.* with Raman or infrared spectroscopy. This problem may be particularly important where the distribution of retained paraffin is not uniform across a tissue section but highly localised to regions of clinical diagnostic interest, such as the epithelia, where important antigens may be co-localised.

The easy elimination of paraffin contamination within tissue sections using these novel SS slides, without a significant disruption in sample preparation workflow, could bring significant advantages in diagnostic pathology. Not only will it facilitate improved protein antigen/biomarker detection, but it will also aid in the elimination of variation in antigen accessibility across a single tissue section. Clinical uptake of SS slides may potentially lead to a more clear-cut diagnostic outcome and a reduction in the antibody concentrations used and associated processing costs. Furthermore, digital archiving of pathology slides is becoming increasingly popular, with the majority of commercial slide scanners being well equipped for reflectance microscopy necessary for non-transparent slides. Aside from the superior paraffin removal properties of SS compared to CaF_2_, the APES-coated mirrored SS are particularly suitable for Raman analysis. At a fraction of the cost to the conventionally used Raman CaF_2_ slides, APES-coated SS provides a much lower Raman background and up to four times signal enhancement leading to an increase in the overall signal to noise ratios as well as imaging speed. As a result these substrates provide a valuable stepping-stone to adoption and integration of Raman technology within the clinical environment.^27^

## Methods

### Slide preparation

Sections of 304L grade super bright polished SS was purchased from Thames Stockholders and cut to the dimensions of conventional histological slides (Thames Stockholders Ltd, Middlesex, UK). Prior to use the protective plastic film routinely provided on highly polished stainless steel was removed to reveal the mirror surface and the residual glue at the edges was removed using ethanol-soaked cotton swabs. SS slides were then washed by dipping in ethanol and coated (silanized) with 2% 3-aminopropyltriethoxysilane (APES) solution (Sigma-Aldrich Ltd, Dorset, UK) for 1 minute using the conventional histopathology protocol.^20^ All other slides including calcium fluoride (CaF_2_) (Crystran, Poole, UK) were cleaned using ethanol prior to use.

### Tissue samples

Rat colonic samples were acquired from a single male Wistar rat obtained from the UCL Biological Services. Euthanasia was conducted using a CO_2_ flow chamber and complete euthanasia was verified using the blink reflex. The colon was then removed and irrigated with ice cold phosphate buffered saline to remove all traces of faecal matter. Multiple resections approximately 5 mm in length were excised and fixed in standard 10% neutral buffered formalin for 24 hours at room temperature. Tissues were then processed into paraffin blocks using standard pathological practice. FFPE human colon and appendix pre-made paraffin blocks were obtained from the UCL/UCLH Biobank for Studying Health and Disease (REC 15/YH/0311).

### Immunohistochemical analysis

Optically transparent 3 μm sections of human appendix were mounted onto Superfrost™ plus charged glass slides and silanized SS for manual IHC staining using a Novolink™ Polymer Detection System (Leica Biosystems Ltd, Newcastle, UK). Deparaffinization was conducted using three sequential 1 minute washes in xylene. Heat-induced antigen retrieval was carried out in a pH 9 Tris-EDTA buffer using a pressure cooker.

MLH1 (Monoclonal Mouse Antibody NCL-L-MLH1, Leica clone ES05) was diluted down to 1 : 200 in TBS Tween, whilst MSH6 (Monoclonal Rabbit Anti-Human MutS Protein Homolog 6, Dako Clone EP49) was used at a 1 : 50 dilution. Both antibody concentrations were optimised by UCL Advanced Diagnostics for use on charged glass slides. Negative control stains were also carried out by omitting the primary antibodies, using DAB only and hematoxylin only.

### Raman microspectroscopy

FFPE tissue blocks were cut at 8 μm thickness and mounted onto silanized SS and CaF_2_ slides. Tissue sections were cut significantly thicker than that required for transmission microscopy to ensure adequate Raman signal during measurements. Sections were incubated at 37 °C overnight to guarantee section adhesion. Through routine use of our APES on SS protocol and increasing experience of tissue handling, we have found that it is not necessary to incubate the sections at 37 °C overnight to guarantee adhesion but for the sake of continuity we continue to employ this practice routinely. Baking the slides at 40–50 °C in the oven for approximately an hour is sufficient, however we have not explored this particular parameter in any detail. We believe that baking the slides for an hour at 60 °C (standard pathology practice) could be too aggressive as SS slides are highly thermally conductive compared to glass.

Sections were then deparaffinized the following day using two sequential 5 minutes washes in fresh xylene and rehydrated in graded ethanol baths (100%, 90%, 70%, 50%) and a final incubation in distilled water. Raman analysis was carried out using a Renishaw benchtop RA816 Raman Biological Analyser (Renishaw plc, Wotton-under-edge, UK) using a 785 nm laser line. A total laser intensity of approximately 160 mW was focused onto the sample through a 50×/NA 0.8 objective. A 1500 l mm^–1^ grating was used to disperse the light providing a spectral range of 0 to 2100 cm^–1^ in the low wavenumber range.

Calibration of the spectrum *x*-axis in absolute wavenumber was done using internal rare-gas emission lines (neon and argon) and in Raman shift using an internal silicon reference to the well-characterised reference peak at 520.5 cm^–1^. Repeatability and reproducibility of response and wavenumber calibrations were tested using a standard internal sample of polystyrene.

The high-resolution rat colonic map was acquired using a 2.8 μm step size with an integration time of 20 seconds producing a Raman map consisting of 81 405 unique spectra. Low resolution mucosa and muscle maps obtained from human colonic tissue mounted on CaF_2_ and SS were at a significantly lower resolution as we were only interested in detecting the paraffin signals. Each map consisted of 336 spectra and were acquired using a 15.7 μm step size with a 15 seconds integration time.

A paraffin reference spectrum was obtained from histological grade paraffin attained from the UCLH department of histopathology.

### Spectral pre-processing and analysis

Cosmic ray removal was conducted using the width of feature and nearest neighbour methods in WiRE 4 software (Renishaw plc, Wotton-under-edge, UK). Baseline correction to a third order polynomial using the modified polyfit method developed by Lieber and Mahadevan-Jansen^28^ and vector normalisation was conducted in MATLAB R2013b (MathWorks Inc., Massachusetts, USA). Pre-processed spectra were then reimported into WiRE 4 and analysed.

Multivariate Curve Resolution-Alternating Least Squares (MCR-ALS) was done using the Renishaw WiRE 4 software utilising the Empty Modelling™ chemometrics feature. This approach decomposes the Raman mapped image into a linear combination of 10 pure component spectra to try and identify regions of paraffin contamination. As a result we were able to obtain spatial information on the regions of contamination in addition to the spectral profile of the contaminating molecular species. Principal Component Analysis (PCA) was also carried our using the Renishaw WiRE 4 software. In short, PCA is a dimensionality reduction approach that reduces a large set of variables into a smaller set that captures most of the variability in the dataset. The first principal component displaying the largest possible variance in the data is often the difference between the tissue material and the backing substrate. The remaining components display the remaining variance in the tissue dataset. Image segmentation was performed using the freely-available Trainable Weka Segmentation (v3.2.5) plugin for Fiji/ImageJ.

### Voltammetric measurements

A dropping electrolyte electrode (DEE) method as outlined by Peretz *et al.* was used to determine the point of zero charge (pzc) of all the metal surfaces tested.^25^

### Scanning electron microscopy

Scanning electron microscopy (SEM) was carried out on Jeol JSM-7401F Field Emission scanning electron microscope, operating at an accelerating voltage of 3 kV. Non-conductive slide substrates (synthetic fused silica and CaF_2_) underwent ion vapour deposition to produce an ultrathin coat of carbon to promote conduction for SEM analysis. SS slides were used without any pre-treatment.

### Atomic force microscopy

Atomic Force Microscopy (AFM) was conducted using a Bruker Innova AFM system (Bruker Corp., USA) to investigate the surface roughness of each slide substrate. Measurements were taken at room temperature in dry air conditions. The spring constant k of the Si OTESPA cantilevers (Bruker Corp. USA) used for all slide substrates was 12–103 N m^–1^ with a resonance frequency of 312–348 kHz. WSxM 4.0 Beta 8.1 freeware software was used to pre-process the images. A flattening filter was applied to correct for abrupt height changes and a global plane filter to adjust for deviations in the image slope.

### Contact angle measurements

Contact angle (CA) measurements were taken using a Drop Shape Analysis System type DSA10-Mk2 (KRÜSS, Optronics GmbH, Hamburg, Germany) equipped with a video camera. A 4 μl droplet of xylene was dropped onto the surface of each slide substrate (CaF_2_, synthetic fused silica, standard glass, SS as well as silanized SS using a micropipette. A time course of CA measurements was obtained by taking the tangent to the drop on each surface. The CA was too small to facilitate static angle measurements. Four time course CA measurements were taken across each surface. Prior to measurements all substrates were incubated for 1 hour in piranha solution (3 : 1 mixture of sulfuric acid (H_2_SO_4_) and 30% hydrogen peroxide (H_2_O_2_)) to remove any surface grease and debris. The silanized SS was fully dried prior to measurement. All measurements were conducted at standard room temperature in ambient air.

## Author contributions

G. M. H. T and K. L. are the principal investigators who conceived the idea for and finalized the manuscript; R. G. designed and carried out the experiments, analysed the data, wrote and compiled the manuscript and figures. R. B. provided helpful discussions and the initial steel substrates during the early stages of substrate testing and development. I. B. was responsible for engineering and physics input. M. R. J. assisted with pathological review and sample acquisition. N. G. and D. O. helped section the tissues, assisted in staining and provided helpful insight into histopathology and staining review. S. C. participated in image analysis using ImageJ. D. J. C. assisted with the electrochemical experiments as well as providing information and feedback on the manuscript.

## Conflicts of interest

Mr Robert Brown is an employee of Renishaw plc where the super mirror steel slides are available for commercial distribution. Dr Katherine Lau no longer works for Renishaw plc and therefore has no competing financial interests. The remaining authors declare no competing financial interests.

Samples of the SS slides can be obtained on request to G. M. H. T.

## Supplementary Material

Supplementary information
