# Supplementary material for: Rapid and complete paraffin removal from human tissue sections delivers enhanced Raman spectroscopic and histopathological analysis
Source: Analyst. 2019 Dec 19;145(4):1499–510. doi: 10.1039/c9an01030k (PMC7677988; doi:10.1039/c9an01030k)

Electronic Supplementary Material (ESI) for Analyst.
This journal is © The Royal Society of Chemistry 2019

ELECTRONIC SUPPLEMENTARY MATERIAL

Supplementary Figure 1

White light images of regions that were Raman imaged from human colonic tissue mounted on CaF_2 and SS slides. **a:** mucosa on CaF_2, **b:** mucosa on SS slides, **c:** muscularis propria on CaF_2, **d:** muscularis propria on SS slides. Raman maps consisted of 336 spectra per map taken using 15 seconds exposure time at 160 mW of laser power using 785 nm and a 15.7 µm step size.

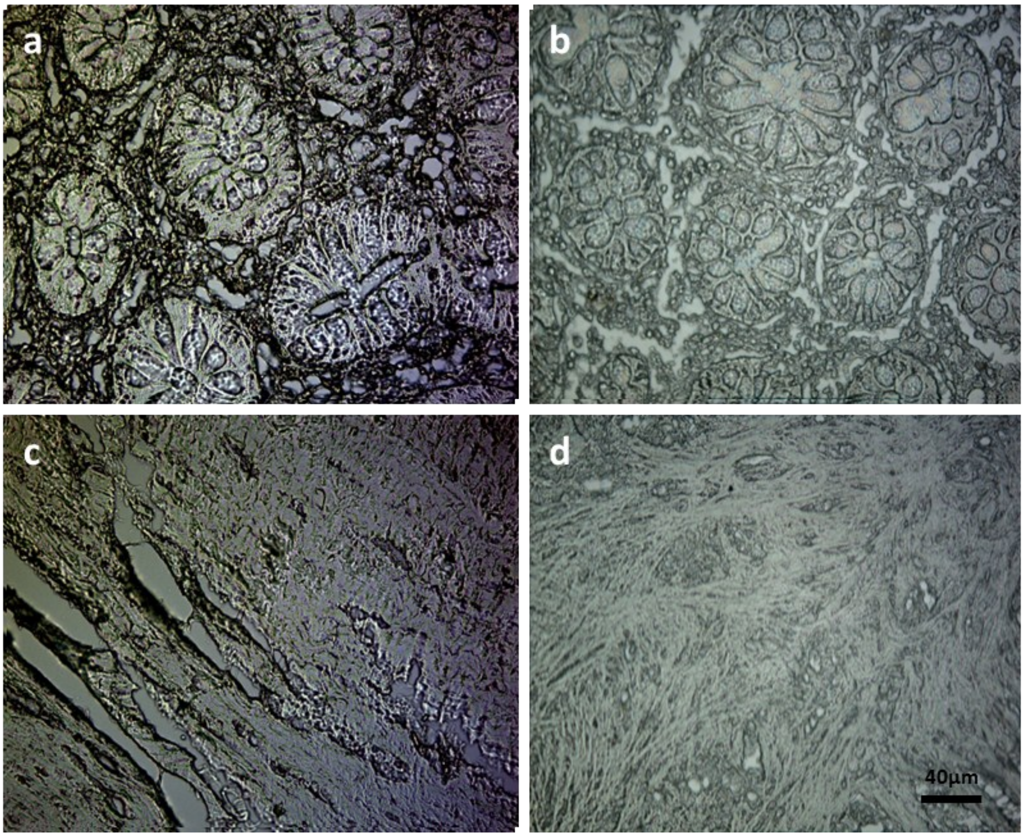

Supplementary Figure 2

First five principal component loading plots from human Raman maps acquired from the mucosal colonic tissue regions mounted onto calcium fluoride (*left column*) and super mirror stainless steel slides (*right column*). Peaks characteristic of paraffin are highlighted with red asterisks.

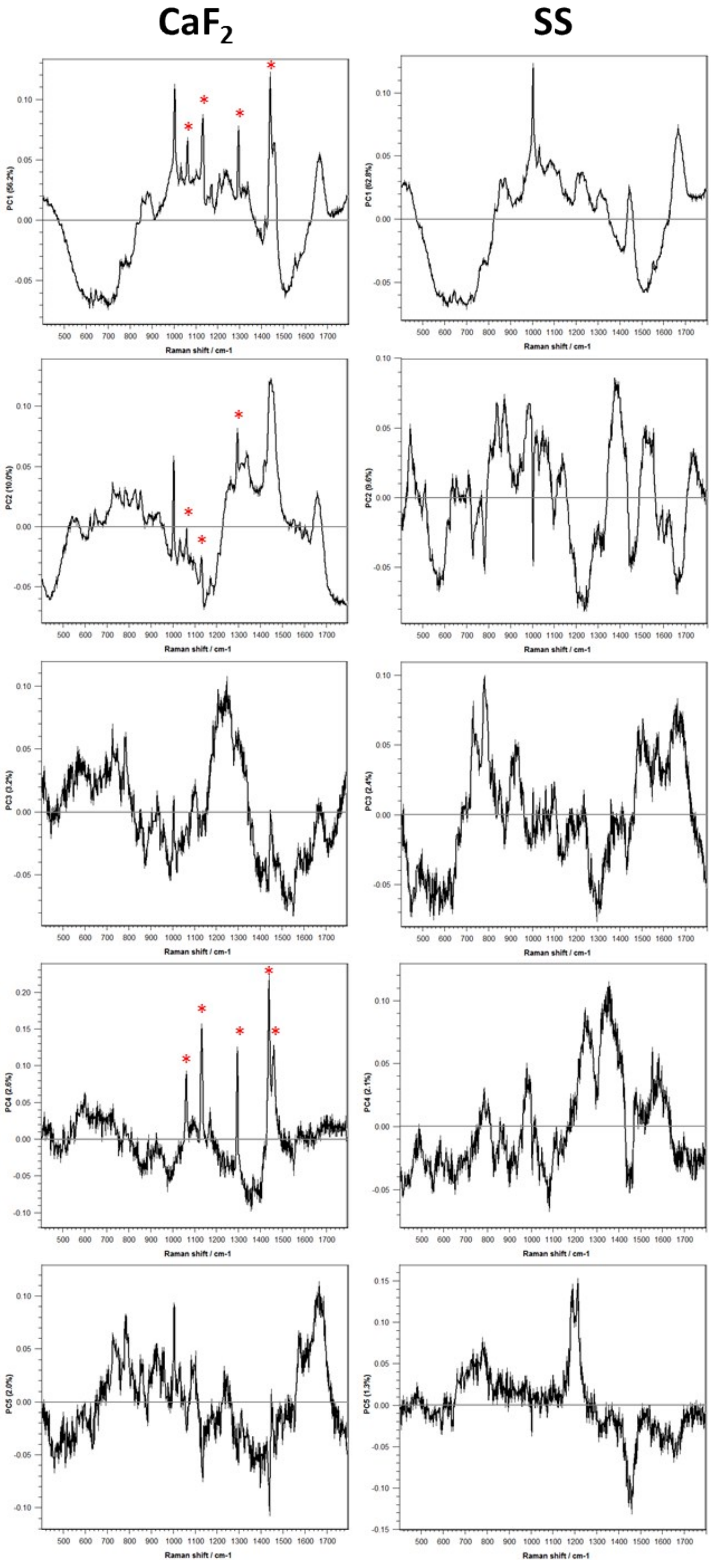

Supplementary Figure 3

First five principal component loading plots from human Raman maps acquired from the muscularis propria colonic tissue region mounted onto calcium fluoride (*left column*) and super mirror stainless steel slides (*right column*). Peaks characteristic of paraffin are highlighted with red asterisks.

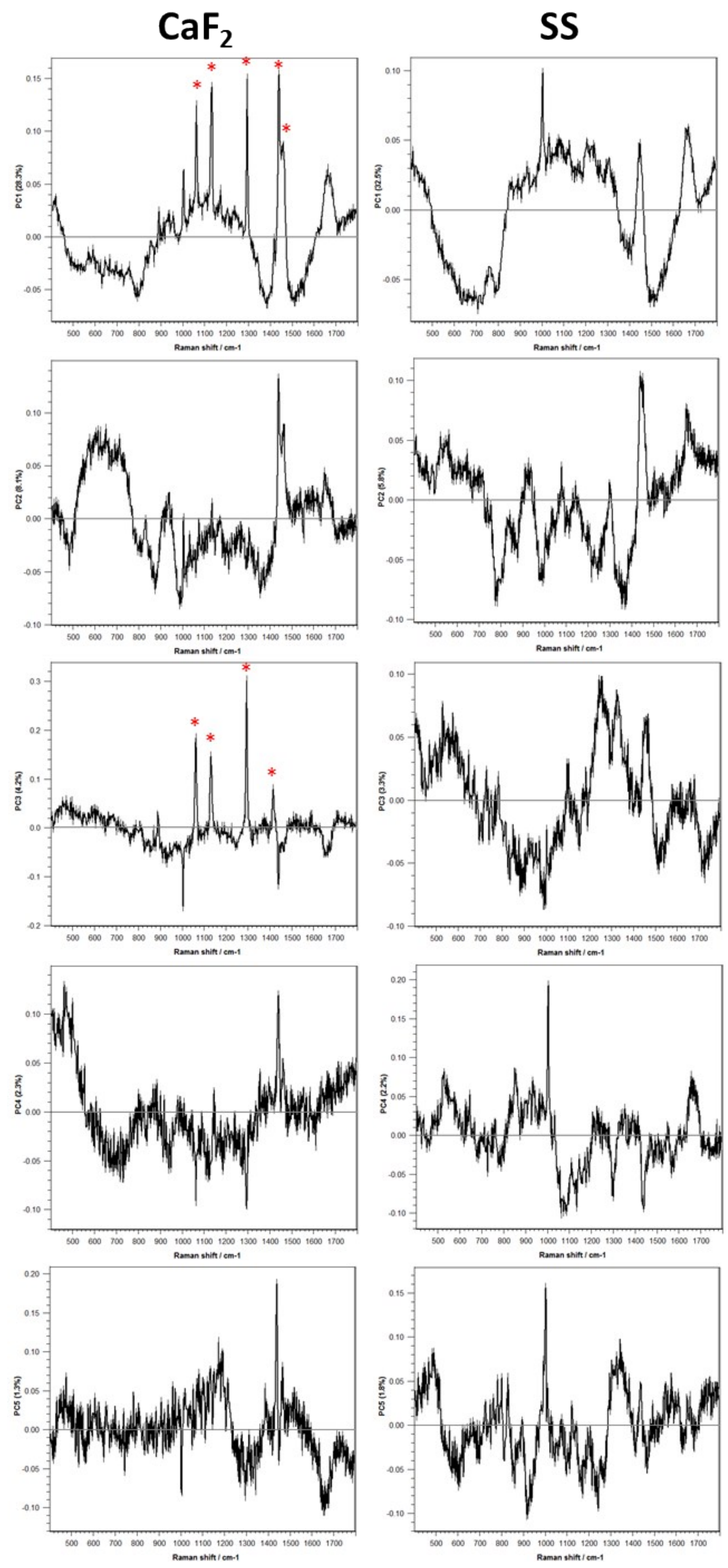

Supplementary Figure 4

Various arrangements of tissue section visualisation during IHC staining intensity evaluation. Non-transparent super mirror stainless steel slides where visualised using epi-illumination (*left panel*). Glass slides were visualised using standard transmission illumination (*centre panel*). To confirm that the IHC staining intensity was not a result of the reflective mirror surface, IHC stained tissue sections mounted on glass were viewed under epi-illumination using a super mirror steel slide placed directly behind the glass slide, such that the mirror surface was in contact with the back of the glass slide (*right panel*).

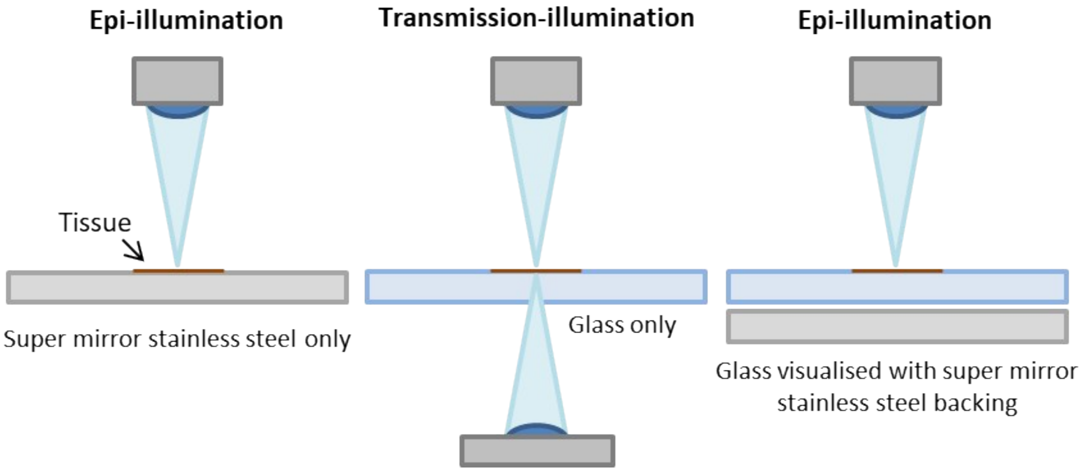

Supplementary Figure 5

SEM images at low (×3,000) and high (×70,000) magnification of the surface features of each slide material. **a:** low magnification synthetic fused silica, **b:** high magnification synthetic fused silica, **c:** low magnification CaF_2, **d:** high magnification CaF_2, **e:** low magnification super mirror steel, **f:** high magnification super mirror steel. Prior to SEM all slides underwent cleaning using a standard degreasing agent trichloroethylene.

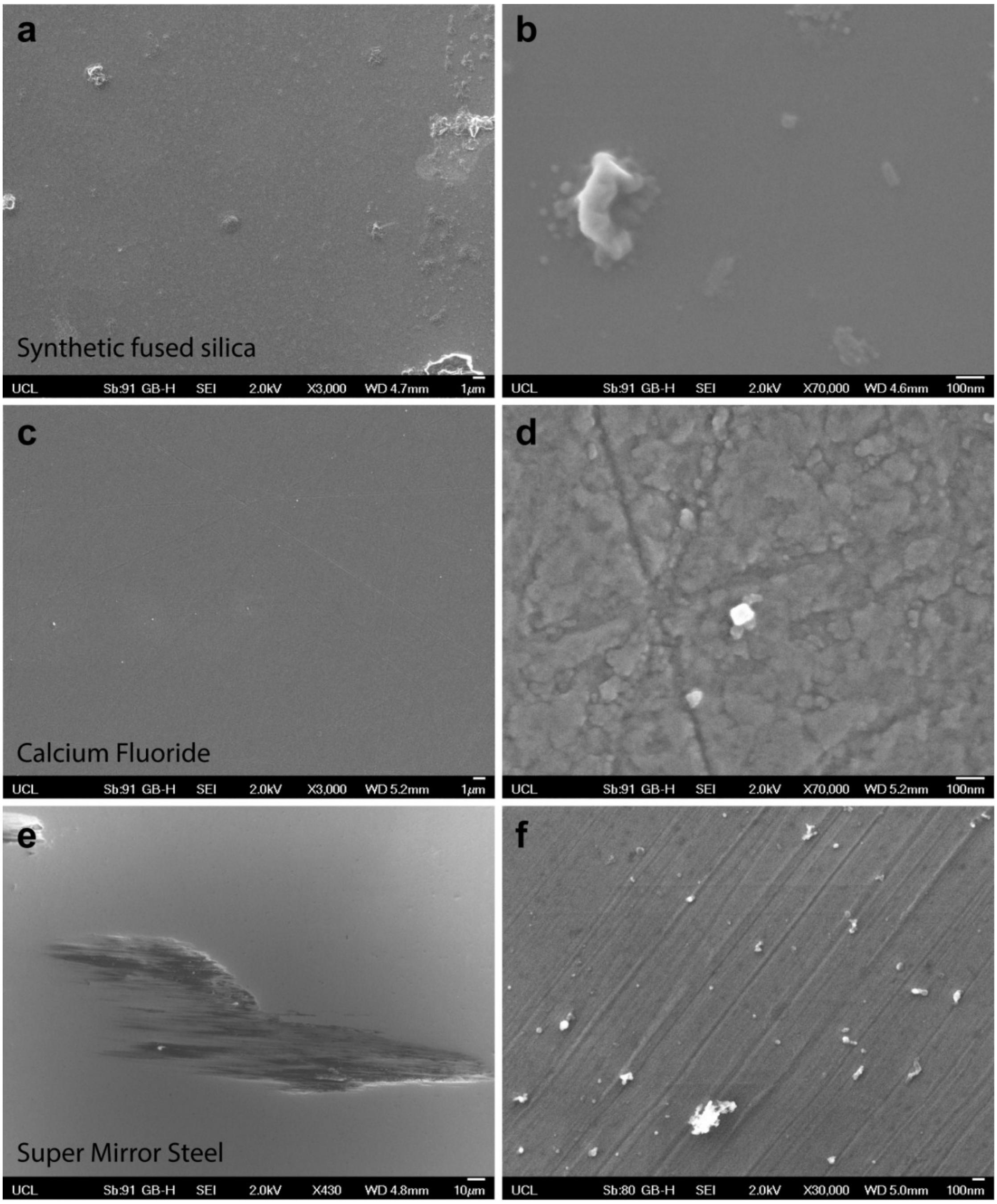

Supplementary Figure 6

AFM images of a 5 µm × 5 µm area on glass, synthetic fused silica, calcium fluoride and SS following cleaning in piranha solution. Average roughness (Ra) values were obtained from the black dashed box regions on the 2D surface representations. Synthetic fused silica and CaF_2 possessed the smoothest surface profiles (0.55 and 0.57 nm) while mirror steel was found to be the roughest (1.81 nm). Surface roughness was derived from regions free of obvious debris defined by the white dashed boxes.

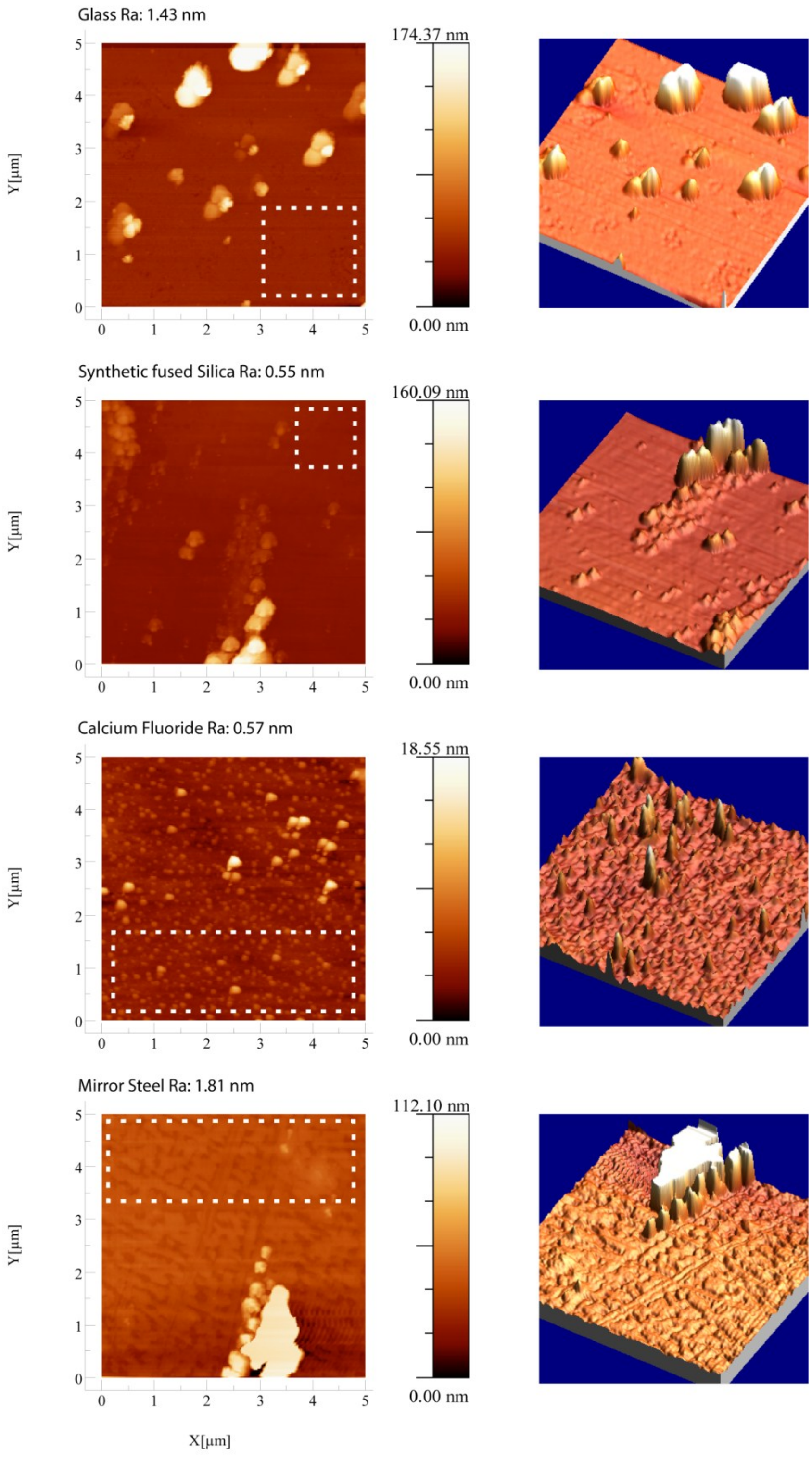

Supplementary Figure 7

Comparison of five original spectra acquired from the surface of mirrored steel, aluminium coated glass and CaF_2. CaF_2 exhibited the greatest baseline drift whereas mirrored steel showed no baseline drift. Aluminium coated glass slides showed an intense broad feature around 750 cm^{-1} attributed to the stretching vibration of the AlO_3 coating.

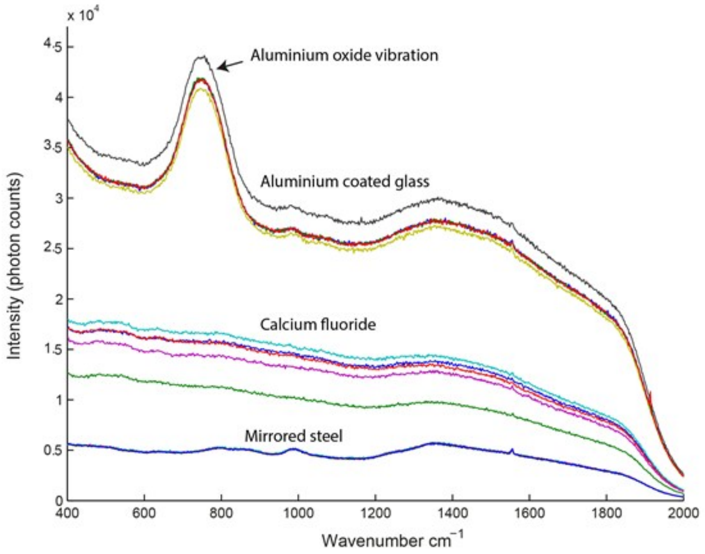

Supplement: Supplementary file 1 [file AN-145-C9AN01030K-s001.pdf]
